# Assessing Public Engagement with Science in a University Primate Research Centre in a National Zoo

**DOI:** 10.1371/journal.pone.0034505

**Published:** 2012-04-04

**Authors:** Mark T. Bowler, Hannah M. Buchanan-Smith, Andrew Whiten

**Affiliations:** 1 Centre for Social Learning and Cognitive Evolution, and Scottish Primate Research Group, School of Psychology, University of St Andrews, St. Andrews, Fife, Scotland; 2 Psychology, School of Natural Sciences, and Scottish Primate Research Group, University of Stirling, Stirling, Stirlingshire, Scotland; Université Paris 13, France

## Abstract

Recent years have seen increasing encouragement by research institutions and funding bodies for scientists to actively engage with the public, who ultimately finance their work. Animal behaviour as a discipline possesses several features, including its inherent accessibility and appeal to the public, that may help it occupy a particularly successful niche within these developments. It has also established a repertoire of quantitative behavioural methodologies that can be used to document the public's responses to engagement initiatives. This kind of assessment is becoming increasingly important considering the enormous effort now being put into public engagement projects, whose effects are more often assumed than demonstrated. Here we report our first attempts to quantify relevant aspects of the behaviour of a sample of the hundreds of thousands of visitors who pass through the ‘Living Links to Human Evolution Research Centre’ in Edinburgh Zoo. This University research centre actively encourages the public to view ongoing primate research and associated science engagement activities. Focal follows of visitors and scan sampling showed substantial ‘dwell times’ in the Centre by common zoo standards and the addition of new engagement elements in a second year was accompanied by significantly increased overall dwell times, tripling for the most committed two thirds of visitors. Larger groups of visitors were found to spend more time in the Centre than smaller ones. Viewing live, active science was the most effective activity, shown to be enhanced by novel presentations of carefully constructed explanatory materials. The findings emphasise the importance and potential of zoos as public engagement centres for the biological sciences.

## Introduction

Research scientists, universities and other research centres are under increasing pressure from funding bodies to demonstrate the societal impact of their research and to engage more closely with the public, who through taxes, donations and other avenues, ultimately provide the funds that make research possible [1]–[4]. Whilst advances in technology or medicine can have obvious benefits to the public, in the field of animal behaviour, ‘impact’ can be difficult to demonstrate. Some discoveries will ultimately reach the popular press or wildlife documentaries and thus have some cultural impact, but many projects will require more innovative strategies to fulfil aspirations of public engagement.

Zoos, the focus of the present paper, have a tradition of science education, often highlighting animal behaviour in active education departments (e.g. the ‘Think Tank’ in the National Zoo in Washington DC, illustrating animal intelligence, exemplified in live studies with apes; http://nationalzoo.si.edu/animals/thinktank/default.cfm). Zoos are also becoming increasingly involved in animal behaviour research, often coupled with public engagement efforts, and in several cases collaboration with universities or research institutes [5]. A prominent example is the Max Planck Institute's enormously productive Wolfgang Köhler Centre, situated in Leipzig Zoo and conducting research on the behaviour and cognition of all four species of great ape (http://wkprc.eva.mpg.de/english/index.htm). The present paper focuses on our own ‘Living Links to Human Evolution Research Centre’ (www.livinglinks.org) in the Royal Zoological Society of Scotland's Edinburgh Zoo, which is both a University research facility focused on primate behaviour and cognition, and a major enterprise in public engagement with science.

Despite academic institutions' large and increasing efforts to engage the public with the sciences, demonstrating that such efforts have measurable effects remains minimal. A surprisingly small literature of such studies exists and the present paper offers an illustration of our own first attempts to quantify some fundamental aspects of public engagement. We note that while progress is being made in auditing and recording public engagement in some UK universities [6], few institutions can demonstrate causal links between their public engagement efforts and such long-term benefits. Measuring the effects of public engagement is not a trivial undertaking, and long-term benefits are inherently difficult to measure. However, levels of public interest in engagement activities and short-term changes in behaviour are easier to gauge, and it is these we focus on here. Implementing recommendations from such assessments should increase the effectiveness of the exhibit and activities, with educational and resource benefits. They also offer the prospect of providing robust evidence of public engagement for funding bodies and university assessment schemes.

Besides the basic observational methods of ethology, we employ methods developed by some zoos and museums to assess their exhibits and education activities. Time spent (“dwell time") and the behaviour of visitors at particular exhibits or activities are often used as measures of interest. For example, Ross and Lukas [7] assessed visitor behaviour at an African ape exhibit at Lincoln Park Zoo, USA, by tracking visitors and recording their behaviour, with the principal goal of using the data in the design of new exhibits. Moss et al. [8] used similar techniques to measure the effect of viewing area size on visit length at an Asian elephant exhibit in Chester Zoo, UK and Anderson et al. [9] used dwell times to measure public interest in presentations at an otter exhibit in Zoo Atlanta, USA. Such data have the potential to determine which factors might increase positive visitor perceptions and experiences.

### The Living Links Research Centre

**Figure 1:**
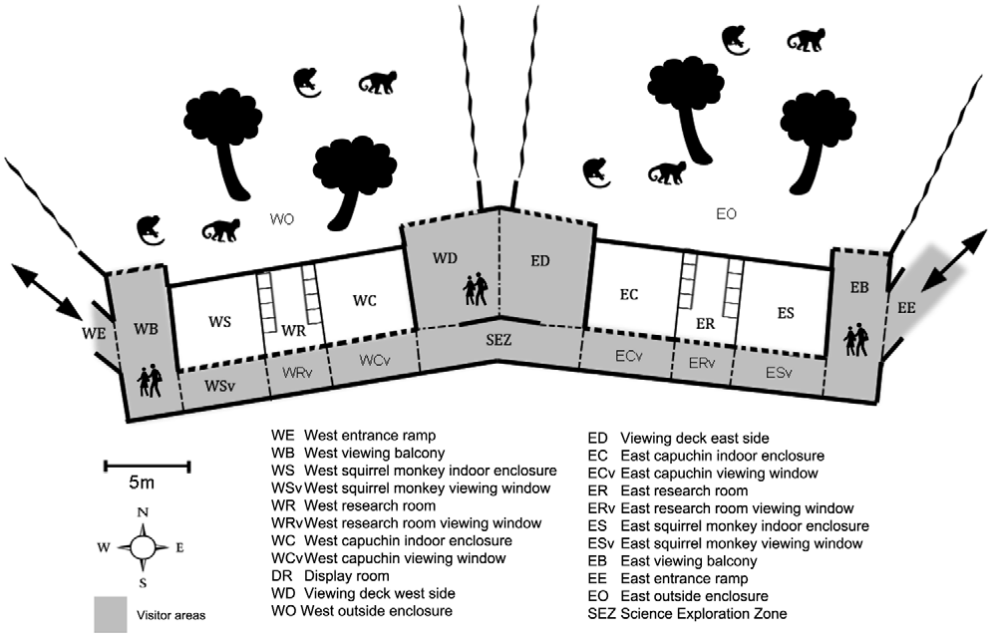
Map of the Living Links to Human Evolution Research Centre showing the visitor zones, research rooms and monkey enclosures (thin dotted lines show how the centre was divided up for data collection and do not represent physical barriers, thick dashed lines show windows or balconies affording views into the enclosures). There are two inside sections to the primate enclosure in each wing, one to which only squirrel monkeys have access (WS and ES) and one to which both capuchins and squirrel monkeys have access (WC and EC).

**Figure 2:**
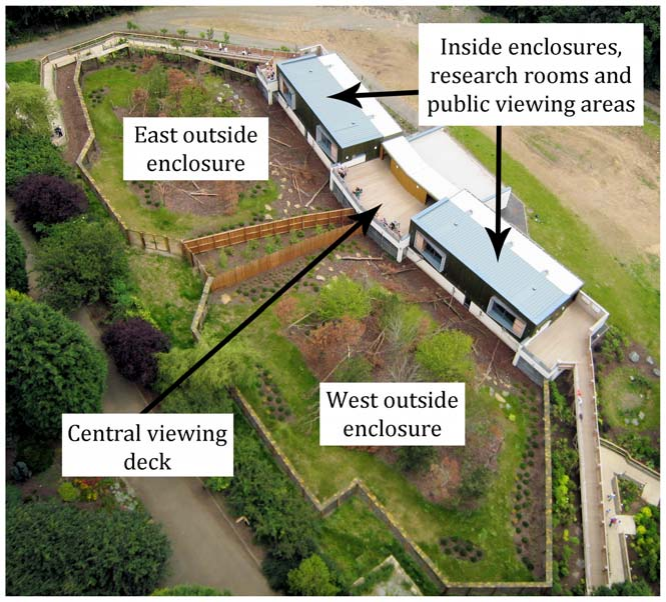
Aerial view of Living Links, showing East and West enclosures, viewing decks for researchers and the public, inner housing (containing research rooms and viewing corridors). *Photo: Stephen Evans*.

‘Living Links’ is at the heart of an innovative and ambitious project that takes on the challenge of engaging the public with behavioural and cognitive research on primates. It is a research facility of the University of St Andrews, built through collaboration between the Royal Zoological Society of Scotland (RZSS) and four Scottish Universities (St Andrews, Stirling, Edinburgh and Abertay). Financed by the Funding Council for the Scottish Universities, its primary function is to provide world-class research facilities for the Scottish Primate Research Group (SPRG) that spans these institutions. It houses two primate species; the brown capuchin (*Cebus apella*) and the common squirrel monkey (*Saimiri sciureus*), living in two mixed-species communities in ‘mirror-image’ ‘east’ and ‘west’ wings (Figures 1 & 2). Further details of housing and husbandry are described in Leonardi et al. [10] and MacDonald & Whiten [11]. Integral to the conception and design of Living Links is its commitment to public engagement. Living Links was built in Edinburgh Zoo, which receives in excess of 600,000 visitors per annum of all ages and backgrounds, and all the research conducted at the Centre is performed in front of the public audience this provides.

**Figure 3:**
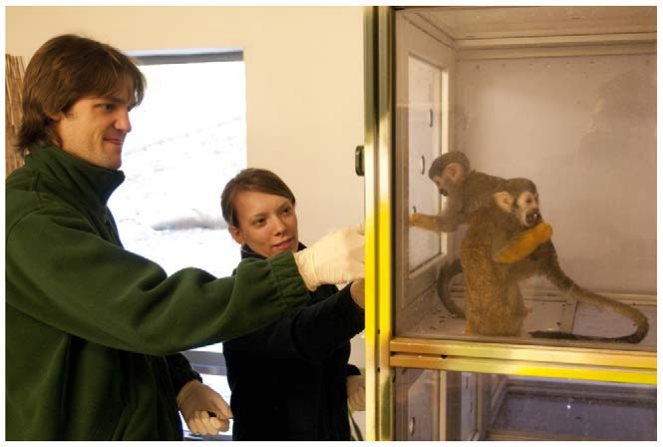
Researchers working with squirrel monkeys in the research room cubicles.

**Figure 4:**
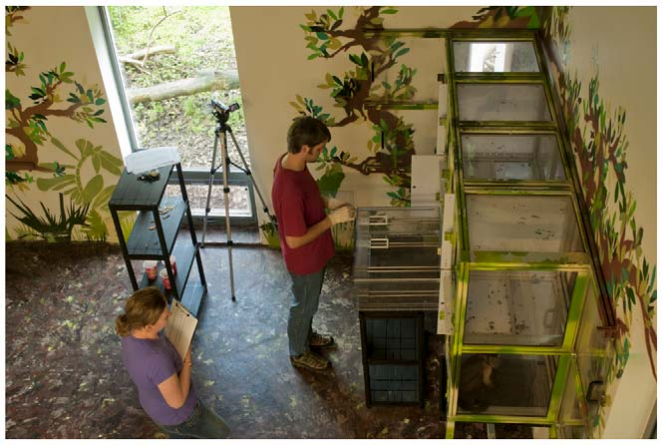
Public view of a research room at Living Links from the visitors' window, with researchers working with capuchin monkeys. Explanatory PowerPoint slides are projected onto the wall above the heads of the researchers, level with the viewing public.

The wide range of studies conducted at Living Links has covered many different aspects of behaviour and cognition, including social learning, communication and prosocial behaviour, with an emphasis on human cognitive and behavioural origins (Macdonald & Whiten [11] provide a detailed overview). Researchers often work closely with monkeys in two dedicated ‘research rooms’ containing ‘research cubicles’, that the monkeys can enter at their own will to participate in behavioural and cognitive experiments (Figures 1 & 3). Visitors can observe ongoing research in these rooms through large windows (Figure 4) and explanation of the research is essential for any deep level of scientific engagement. The presentation of such explanations, and the facilitation of two-way engagement between researchers and the public, without compromising the research itself, is expected to be key to the success and impact of the initiative.

Between June 2009 and September 2010, we repeatedly assessed public interest in the research and educational materials presented at Living Links. We recorded changes in visitor behaviour and dwell times in relation to research activities, and as new educational and explanatory materials were installed. We also tested the expectation that people will be more interested (and dwell longer) at certain parts of the exhibit if they are provided with suitable explanations about what is happening, and why.

### Public Engagement: (i) Information boards and other educational materials

Visitors enter Living Links from either the east or west, and first encounter views into the outside and inside living quarters of the monkeys. Visitors then see into the research rooms, each with a projector showing explanatory slides of the research, or recorded videos when research is not running. The corridor continues to a central area that latterly became the Science Exploration Zone (SEZ), from which visitors can either continue through the inside sections or turn out onto a large viewing balcony overlooking the outside enclosures. Walking through the Centre without stopping or visiting the balconies takes around two minutes.

**Figure 5:**
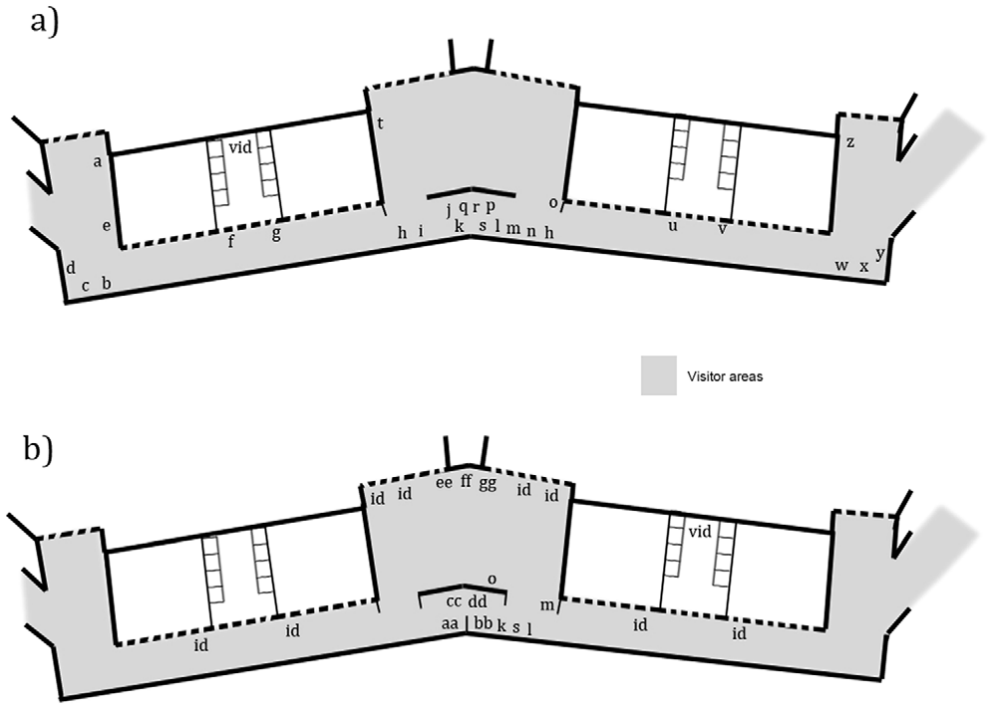
The Living Links Centre showing a) the positions of information boards and barriers in June 2009 and b) the positions of additional information boards and barriers added or moved between 2009 and 2010. Thick dashed lines show windows or balconies affording views into the enclosures. The information boards corresponding to the codes are described in Table S1.

**Figure 6:**
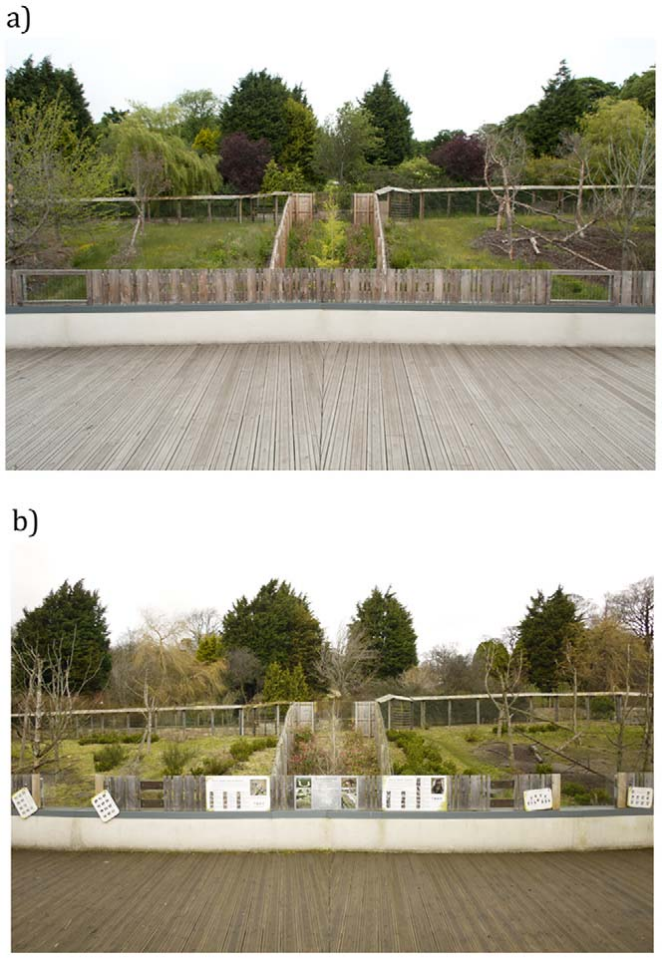
Central viewing deck at Living Links before and after the addition of multiple information boards (*id*, *ee*, *ff* and *gg* in Table S1).

At its opening in 2008, Living Links had 28 information boards, projected videos and three interactive educational activities displaying further information about Living Links and research on primates (Figure 5a; Table S1). A further 16 such elements were added between October 2009 and July 2010, using guidance from published assessments of zoo signage [12]–[15] to design and position the displays (Figure 5b; Table S1). Many of the new materials added were aimed at a younger audience than the original signs, which had been designed to provide a relatively complete explanation of the Centre from the outset. Additionally, mesh panels on the viewing balconies were replaced with toughened glass panels, allowing children and wheelchair-users much clearer and more frequent viewing opportunities, and giving the viewing balconies a more ‘open’ and inviting look (Figure 6).

**Figure 7:**
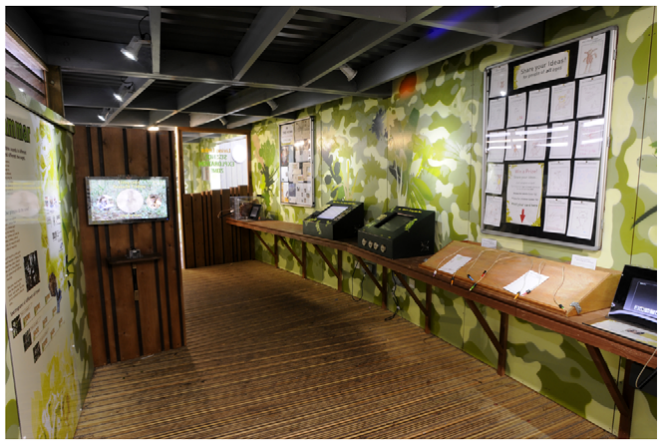
The Science Exploration Zone (SEZ) at Living Links, after renovation in 2010, with information boards and interactive materials (from left to right, codes from Table S1): *k, bb, cc, j, q, r, n, dd*.

**Figure 8:**
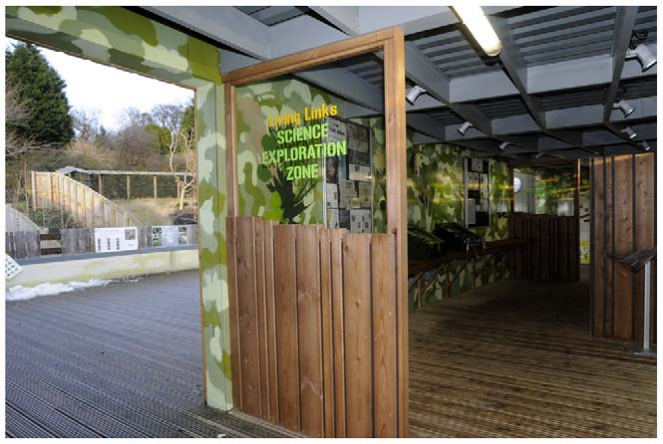
One of the newly installed new dividing walls of the Science Exploration Zone (SEZ) at Living Links, after renovation in 2010, showing how visitor flow might be encouraged out to the outside central viewing deck on the left.

Between the 2009 and 2010 summer seasons, small dividing walls were added to the Science Exploration Zone (SEZ) to more clearly designate this space and its role. The SEZ was re-modelled along the lines of a traditional ‘science centre’ presenting information boards and several hands-on interactive learning activities (Figure 7). This area was designed to be inviting particularly to youngsters, and also change visitor flow though the exhibit – transforming what was previously a wide corridor into a partitioned ‘room’ to be entered by those particularly interested. The principal flow of visitors was now expected to involve walking onto the central outdoor viewing balcony, with a subset visiting the SEZ at this point, or after spending time on the viewing balcony (Figure 8). In the SEZ two new interactive computer displays were added to the two that already existed, together with experimental ‘puzzle boxes’, originally used in studies of social learning in chimpanzees [16], but here adapted to attract the interest of children and their parents. The SEZ was also used for face-to-face science communication during the Edinburgh International Science Festival, participatory psychology experiments with visitors, and for other ephemeral activities.

### Public engagement: (ii) Viewing and participating in research activities

Observational research can occur at any time of day in Living Links, but experimental research, especially when conducted in the research rooms, is generally scheduled for late morning or mid-afternoon. All can be directly observed by the public. When experiments took place, and when available, the ‘Science Communication Officer’ (MB) explained and discussed the research with visitors at the research room window. After the afternoon research session, seasonal daily talks on the research activities were given by the Living Links animal keepers, the Science Communication Officer or the Zoo's Education Department staff. These talks were often accompanied by a ‘scatter feed’ for the monkeys, or hands-on demonstrations of research apparatus.

In conjunction with the Zoo's own education programme, school groups and summer school classes of all ages are brought to Living Links where they observe animal behaviour and collect data on live monkeys. We encouraged similar activities by regular Zoo visitors during peak times via project sheets offered in leaflet dispensers positioned in key areas of the Centre.

### Ethics Statement

The study was a purely observational study of Zoo visitors and was approved by the ethics committee of the University of Stirling.

## Methods

To assess interest in the live research and public engagement activities in Living Links, including the use of educational materials, we used focal individual continuous sampling [17] to measure visitor movement through the exhibit, designating a series of visitor ‘zones’ (Figure 1) and recording the time spent in each section. The first adult visitor (estimated to be over 18 years old) entering Living Links at the start of each sampling period was selected as a focal individual and followed until they left the exhibit, including time on the entrance and exit ramps. The gender and estimated age of the focal individual were recorded, along with the size and age-sex composition of their group. Group members were defined as ‘all individuals that the focal individual both entered Living Links with and interacted with during their visit’. No other details that could identify the visitor were recorded, in order to maintain anonymity and privacy. Between two and 16 focal follow samples were collected each sampling day. Due to ethical considerations, data were not collected on children in this way.

We recorded the length of time the focal individual spent interacting at each information board, video or interactive activity in the exhibit, defining ‘interacting’ as stopping within 2 meters of the item and oriented towards it from more than 1 second. Data were collected on both weekdays (*N* = 65 follows) and weekends (*N* = 148 follows) in 2009, but preliminary analysis showed that visitor behaviour differed in these two conditions (visits being 12% longer at weekends). Since research sessions with the monkeys were conducted mainly on weekdays, and were one of the activities we were most interested in assessing, we collected data exclusively on weekdays (*N* = 70 follows) in 2010 to compare years. The data used to compare years were restricted to those collected in June, July and August of each year. Data were collected on 14 ‘no rain’ research days in each year to reduce the effect of weather as a confounding variable. Similarly, to control for differences in behaviour between visitors entering from the east or west, we selected focal individuals entering only from the more commonly used west ramp. Scan samples [11] were also made between each focal follow by walking through the Centre, recording the presence of monkeys on view from each visitor zone, and the number and behaviour of all people in each zone using the categories ‘watching monkeys’, ‘walking’, ‘reading boards’ and ‘other’. Mean daily temperatures were taken from the nearest available weather station with full available records; Lochgelly, Fife [18], approximately 20 km from the zoo.

In addition to the scans and the full focal individual follows, we included 250 short focal individual follows. These used the same method as the longer follows, but were restricted to the indoor visitor zone in front of the west research room window. Here we manipulated the conditions to assess changes in behaviour during different activities in the research room. Data were collected under the following conditions: No activity, no videos showing (*N* = 50); videos presented on a continuous 3-minute loop (*N* = 50); videos selectable via touch sensitive buttons (see Table S1) (*N* = 50); live research without an explanatory PowerPoint slide (*N* = 50); and live research with a brief explanatory PowerPoint slide and an appropriate photograph of the research (*N* = 50). These data were collected during short 15-minute research periods between October 2009 and July 2010, with research periods for each condition matched as closely as possible for time of day and time of year.

### Data analyses

We tested data for normality using the Kolmogorov-Smirnov test, and where data were not normally distributed, we used non-parametric statistics and report medians and inter-quartile ranges, as well as means. Where data were normally distributed, parametric tests, means and standard error of the means are provided.

## Results

### Visit Durations

The addition of engagement elements was associated with an increase in mean weekday visit durations measured in focal follows, between summer 2009 (mean = 9 min 46 s, median = 9 min 27 s) and summer 2010 (mean = 12 min 13 s, median = 11 mins 21 s) (Mann-Whitney U test: *U* = 2965, *N*_1_ = 65, *N*_2_ = 70, *P* = 0.002). The way this time was spent within the different visitor zones did not change uniformly. Time spent on the viewing ramps, balconies and the inside squirrel monkey viewing windows increased, whilst average time spent in the SEZ decreased and time at the inside capuchin and research windows did not change significantly (Table 1).

**Table 1:** Dwell times in visitor zone categories at Living Links.

	*N*	Mean + SE dwell time per zone category (s)						
		Ramps	Capuchin Windows	Science Exploration Zone	Squirrel Windows	Research Windows	Outside Balconies	Visit length
2009	65	129.4+5.0	126.4+12.5	49.5+6.4	115.2+11.9	49.2+6.2	116.0+11.0	**585.8 + 217.5**
2010	70	145.6+5.7	147.4+16.5	38.6+7.2	150.6+11.8	47.9+6.7	203.3+19.8	**733.3 + 280.8**

	*N*	Median+IQ dwell time per zone category (s)						
		Ramps	Capuchin Windows	Science Exploration Zone	Squirrel Windows	Research Windows	Outside Balconies	Visit length
2009	65	122+104–141	93+58.5–163.5	25+14–84	88+58–158	30+17.5–64	108+56.5–149.5	**576 + 438.5–672.5**
2010	70	136+111.5–163	104.5+61.5–193.5	2+2–53.5	121+92–197	28+16.5–58	154.5+91.5–265	**680 + 551.5–929**
Mann-Whitney U		2836.5	2430.5	1409	2910.5	2157	3135	2965
*P*		*P = 0.013	*P* = 0.493	* *P*<0.001	* *P* = 0.005	*P* = 0.603	* *P*<0.001	* *P* = 0.002
Change from 2009 to 2010		Increased	No change	Decreased	Increased	No change	Increased	Increased

On reaching the SEZ, more people turned out onto the central balconies in 2010 compared with 2009, although these people still passed through the end part of the corridor to access the central balcony area. The number of people spending less than ten seconds in the area thus increased significantly from 16.9% in 2009 to 55.7% in 2010 (Chi-square test: χ2 = 21.75, *P*<0.001). This may be a result of more people being diverted out by the new partitions introduced to delimitate the SEZ (Figure 8), or of their choosing to go outside into an area made more inviting by new information boards and glass viewing panels (Figure 6).

Weather differences might be expected to affect dwell times, with the expectation that dwell times would be longer in outdoor exhibits in warmer weather. However, this is unlikely to explain observed changes, because daily temperatures for weekday follows in 2010 (median 15.2°C) were actually slightly lower than in 2009 (median 16.3°C) (Mann-Whitney U test: *U* = 1282.5, *N_1_* = 65, *N_2_* = 70, *P*<0.001). Furthermore, there was no correlation between temperature and visit durations in either 2009 (Spearman rank correlation: rs = −0.188, *N* = 65, *P* = 0.067) or 2010 (Spearman rank correlation: rs = −0.040, *N* = 70, *P* = 0.370).

Focal follow data showed that total visit duration was positively correlated with visitor group size (Spearman rank correlation: *r*_s_ = 0.208, *N* = 135, *P*<0.015), and that visitor group size did not differ between years (ANOVA: *F*_1,133_ = 0.205, *P* = 0.651). On weekdays, 64.4% of groups (group size *X*+SEM = 3.2+0.17, *N* = 135) contained children (estimated as under 16 years old), which did not vary between years (Chi-square test: *χ* ^2^ = 1.958, *P* = 0.208). There was no difference between the visit lengths of groups containing children under 16 and groups with no children (Mann-Whitney U test: *U* = 2041.0, *N*_1_ = 48, *N*_2_ = 87, *P* = 0.829).

From the scan samples, the mean number of people in Living Links at any point in time during data collection periods across both years was 29.4 (SE 1.33, range 3–78). The median number of people in Living Links in these periods increased from 20.5 in 2009 to 29 in 2010 (Mann-Whitney U test: *U* = 3524.0, *N_1_* = 60, *N_2_* = 84, *P*<0.001).

### Presence of monkeys

Since visitor dwell times increased particularly in the outside sections of Living Links between 2009 and 2010, we compared the average number of monkey ‘groups’ (taking any members of one species in either the west or east mixed-species enclosures as a ‘group’) visible to visitors in the outside enclosures during scan sampling sessions in 2009 and 2010. There was no difference between the number of monkey groups outside during data collection in 2009 and 2010 (Mann-Whitney U test: *U* = 2441.5, *N*_1_ = 60, *N*_2_ = 84, *P* = 0.740), indicating this was not the reason for changes in visitor behaviour.

### Information board reading and time at interpretive materials

On weekdays through 2009 and 2010, 8.9% of visitors (including children) in scan samples (*N* = 144 scans, containing 4227 visitors) were recorded reading information boards or using interactive materials at Living Links. From this we can estimate that the average visitor, across the two years, spent around 59 seconds of an 11 minute 2 second visit reading signs or engaging with interpretive materials. However, despite the addition of new information boards and interactive activities, the average number of boards and other interpretive materials looked at per adult visitor (recorded in focal follows) did not change significantly between 2009 (median+IQ = 1+0–2) and 2010 (median+IQ = 1+0–3) (*U* = 2397, *N*_1_ = 65, *N*_2_ = 70, *P* = 0.579). The mean time (seconds) spent at boards and other interpretive materials more than doubled between 2009 (mean + SE = 24.1+4.55, *N* = 65) and 2010 (mean + SE = 62.31+12.48, *N* = 70) but variance was high and the difference between time (seconds) spent interacting with materials in 2009 (median+IQ = 8+0–43, *N* = 65) and 2010 (median+IQ = 12.5+0–101.5, *N* = 70) was not significant in a U-test (Mann-Whitney U test: *U* = 1913.0, *N*_1_ = 65, *N*_2_ = 70, *P* = 0.211).

These statistics are much influenced by the fact that focal follows showed that just under 36% of weekday visitors did not stop at any boards or interactive activities, a proportion that did not change significantly between 2009 and 2010 (Chi-square test: *χ* ^2^ = 0.16, *P* = 0.689). Informal observations and the differences between the medians and the means suggest that the two thirds of visitors that *did* choose to engage with boards, videos or interactive computer games sometimes spent quite long periods doing so. If we exclude visitors who did not engage with materials from the analysis, the time spent (in seconds) in engagement did increase significantly, tripling between 2009 (median+IQ = 20+8–50, *N* = 43) and 2010 (median+IQ = 64+14.5–130, *N* = 44) (Mann-Whitney U test: *U* = 1302.0, *N*_1_ = 43, N_2_ = 44, *P* = 0.002), although the number of boards looked at did not show a significant increase (2009; median+IQ = 2+1–3, *N* = 43, 2010; median+IQ = 3+1–4, *N* = 44)(Mann-Whitney U test: *U* = 1143.0, *N*_1_ = 43, *N*_2_ = 44, *P* = 0.850). These results suggest that what changed between 2009 and 2010 was that the two thirds of visitors prone to some level of engagement showed on average longer dwell times, indicating higher levels of interest in the materials added between years.

The presence of children under 16 years old in a group had no effect on the number of elements looked at by focal individuals (Mann-Whitney U test: *U* = 1810.0, *N*_1_ = 48, *N*_2_ = 87, *P* = 0.187) nor on the time spent looking at them (*U* = 1843.0, *N*_1_ = 48, *N*_2_ = 87, *P* = 0.449).

**Figure 9:**
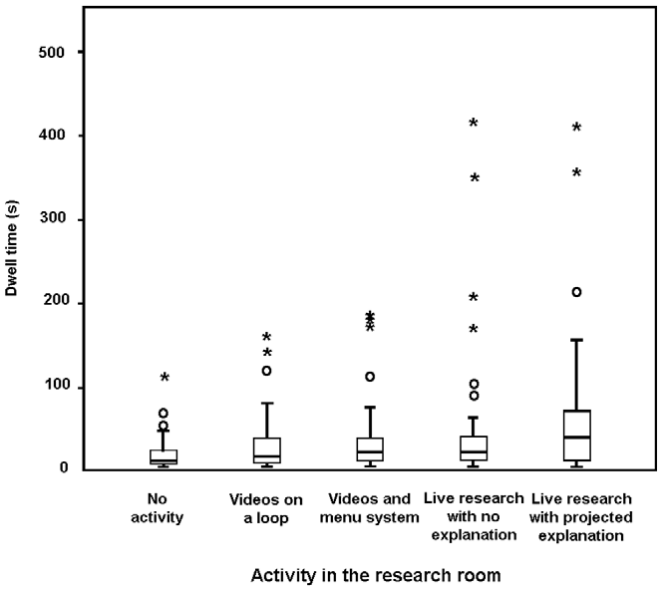
Visitor dwell times at the research windows during public engagement activities: No activity or videos showing (*N* = 50); videos presented on a continuous 3-minute loop (*N* = 50); videos selectable via touch sensitive buttons (see Table S1) (*N* = 50); live research without an explanatory PowerPoint slide (*N* = 50) and live research with a brief explanatory PowerPoint slide and an appropriate photograph of the research (*N* = 50). Central bar is median, box indicates the upper and lower quartile for the middle 50%, and whisker the upper and lower 25%. Asterisks and circles show outliers.

Visitor dwell times at the research window during short follows varied depending on the activity in the room (Kruskal-Wallis test: *H*_4_ = 21.53, *P*<0.001), with live research activities being the most watched (Figure 9). Using Mann-Whitney U as a selective *post hoc* test to identify differences between the two ‘non-research period’ video conditions, we detected no difference in visitor dwell times when videos were presented on a 3-minute loop to when visitors could select videos from a menu (Mann-Whitney U test: *U* = 1367.0, *N*_1_ = N_2_ = 50, *P* = 0.420). However, during research sessions, visitors spent longer at the windows when a descriptive PowerPoint slide was projected onto the rear wall than they did when no further information was projected (Mann-Whitney U test: *U* = 1534.5, *N*_1_ = *N*_2_ = 50, *P* = 0.050).

## Discussion

Our initial attempts to quantify some basic aspects of the public's response to our science engagement offer some encouraging findings, along with others that, at first sight, are relatively discouraging. Amongst the latter are the findings that about one third of visitors do not attend to engagement elements, and that the average visitor will engage with only one of the numerous elements we have incorporated into the Centre. However it should be noted that we were unable to determine how many of our visitors were visiting Living Links for the first time; many could have been return visitors who had read some of the signs before; for example in 2009, 14% of visitors were members of the Royal Zoological Society of Scotland, who gain free entry to the zoo and might reasonably be expected to have visited Living Links on previous occasions. Also important in interpreting these results is the fact that the profile and motivation of people visiting a zoo are very different from those who choose to visit a science centre or science fair, for example. The latter are likely to be strongly biased towards an educated, middle-class clientele who are visiting intentionally to engage with science. Zoo visitors, numbering over 600,000 per annum in the case of Edinburgh Zoo - more than those attending all the Scottish science centres put together - cover almost the entire educational and socioeconomic spectrum and will also include those visiting the zoo for an entertaining day out, not to grapple with science [19]. Even if up to a third of visitors showed little or no interest in our science engagements efforts, this should not be too surprising or disheartening.

The implications of the demographic profile of zoo visitors, alluded to above, amount to a context that casts a much more encouraging light on the positive aspects of engagement we documented. The average visit durations, of over nine and over twelve minutes respectively in the two years of our study, are regarded as being noticeably high by zoo staff, and it is encouraging that the addition of multiple new engagement elements in the second year was accompanied by a significant increase in visit duration. Although this may in principle be due to a number of factors, we showed that it was unlikely to be caused by the weather, and note instead that it is consistent with the aspirations of the programme of additional engagement activities in the second year. It is possible that the increase in variety and availability of information and interactive materials at viewing points overlooking monkeys and research studies stimulated more interest in the monkeys and increased dwell times at viewing areas. The exception was in the SEZ where visitor dwell time was significantly reduced. Certainly interest in live research was increased by the provision of a description of the experiment and objectives of the research. People engaged for longer when they were better informed, and during informal observations, parents were often seen explaining the research to their children by reading and interpreting the information and then providing their own commentaries on what was happening in the research room. This demonstrates that our visitors often have an interest in the science they are seeing in front of them, and are prepared to invest time in learning more and passing on this knowledge.

Interestingly, these effects were seen most obviously in the sub-sample (2/3^rd^) of visitors who were prepared to engage at all. For them, the time spent in engagement, although still very modest on average, tripled in the year that the additional engagement elements were introduced. This was not a small subsample, but represented two thirds of the visitors. If we multiply the modest time they spent in engagement by our conservative estimate that half the zoo visitors pass through Living Links (i.e. over 300,000 per annum), this adds up to over 5,000 hours of active engagement with signs and interactive materials a year, across a very broad demographic sample - and note this does not include time spent watching the monkeys or the research itself. Note also that in this first study, we did not collect data on children's responses. This is a significant omission given that so much of the science engagement material in Living Links is directed at them and a current study is designed to gain relevant data for this sample through different methodological approaches.

We note also that the above picture is based largely on individual focal sampling of a very large visiting population. Our scan sampling alludes to what is apparent on an average, good-weather summer visiting day, when many of the engagement elements studied here will be attended to by multiple individuals, with some visitors even waiting their turn, or being involved as a small group. Focusing on mean and median values of number of visitors at any one time point may hide a large number of visitors engaging with our information and interactive materials for long periods. Additionally, the ‘short-follows’ study at the main research room windows provided quantitative evidence that watching live science in action was the most engaging activity we presented here, and interest was significantly enhanced by relevant explanation in the form of a power-point slide projected on the large back wall of the research room.

Studies employing similar methods to ours are generally concerned with enclosure design in zoos and in maximising visitors' experiences (e.g. [9],[20]–[22]); indeed it is reported that most zoos have active visitor research [23], but publication of results appears relatively rare. More recently, a few studies have begun to assess how education goals are met [7],[24]–[27], but these publications, in the specialist zoo and museum literature, do not appear to address the wider implications and importance of this kind of research. As zoos are starting to realise the wider scientific and social importance of their research into visitor behaviour, universities are realising the need to engage with the public and are looking for means to do so.

### Zoos as ‘public engagement centres’ for the biological sciences

In the UK, science and discovery centres and museums receive around 19.5 million visitors a year, but to our knowledge, only two centres in Europe offer large numbers of visitors the chance to observe real, university-lead research in animal behaviour; the Wolfgang Köhler Primate Research Center in Leipzig Zoo, and the Living Links Centre described here. As noted above, zoo visitors often outnumber visitors to science centres, and are likely to come from a wider range of backgrounds and interests. Visitors to science centres are likely to be inherently interested in science; zoo visitors likely have a wider range of interests. This not only makes zoos more encompassing venues for public engagement, but may also make them more suitable venues at which to measure its potential effects. Zoos present opportunities for public engagement projects of various scales, in a real-life context for researchers working on animal behaviour. Zoos often have their own perspectives and expertise in working with the public that university researchers can draw on when expanding their public engagement activities, whilst zoos can benefit much from the input of research scientists, constantly updating, augmenting and diversifying high-grade educational activities and materials.

## Supporting Information

Table S1 **Descriptions of Information boards and interactive displays at Living Links.**
(PDF)
