# Supplementary material for: Assessing Public Engagement with Science in a University Primate Research Centre in a National Zoo
Source: PLoS One. 2012 Apr 4;7(4):e34505. doi: 10.1371/journal.pone.0034505 (PMC3319593; doi:10.1371/journal.pone.0034505)
Supplement: Table S1 — Descriptions of Information boards and interactive displays at Living Links. (PDF) [file pone.0034505.s001.pdf]

Table S1.

Information boards, videos and interactive computer displays				
**Location Code**	**Name**	**Type**	**Description**	**Date Installed**
a,z	Living together	Information board	Explaining that the squirrel monkeys and capuchin monkeys live in a mixed species group	From opening
b,w	Map	Information board	A map of the Living Links (2 versions west and east)	From opening
c,x	Living Links Research Centre	Information board	General background information on the centre	From opening
d,y	The Scottish Primate Research Group	Information board	Introducing the Scottish Primate Research Group	From opening
e	Opening plaque	Plaque	A plaque commemorating the opening of Living Links by Jane Goodall	From opening
f,u	Research Room	Information board	Explaining the purpose and function of the research rooms	From opening
g,v	Current Research	Notice board	Short descriptions of current research activities taking part in the research rooms	From opening
h	Seeing double	Information board	Explaining Living Link's 'mirror-image' design for research (2 versions west and east)	From opening
i	Notice board	Notice board	Used for instructions and explanations for research activities running at Living Links	From opening
j	Notice board	Notice board	Clippings of recent Living Links news items from the popular press	From opening

k	Grammar	Information board	Grammar in primates, based around SPRG research on communication in primates	From opening
l	Meaning	Information board	Based on SPRG research on communication in primates (accompanies ‘s’)	From opening
m	Language	Information board	Language in primates, based around SPRG research on communication in primates	From opening
n	Notice board	Information board	Used in 2009 to display profiles of the main people working at Living Links, and in 2010 for details of current research in progress outside the research rooms.	From opening
o	Evolutionary Tree	Information board	Diagram of the ‘tree’ of Old world primates, including humans	From opening
p	Notice board	Notice board	(‘Who's who’ at living links and current research notices)	From opening
q	Interactive 1	‘Interactive’	A computer screen and microphone producing sonograms of visitors voices and challenging them to reproduce primate calls	From opening
r	Interactive 2	‘Interactive’ game or display	A touch screen quiz about chimpanzee communication	From opening
s	Interactive 3 –	‘Interactive’ game or display	Six buttons producing monkey alarm calls for various predators with photographs and captions (accompanies ‘l’)	From opening
t	Brown capuchins	Information board	General information about capuchin monkeys and their use of tools	From opening

vid	Video	AV projector	<u>West</u><br>Running powerpoint slides from opening until 29-8-09 when replaced by videos on a loop until 2-12-09 when a touch sensitive menu was on the window to select from various new films	From opening
			<u>East</u><br>Running the most important videos on a loop explaining the purpose of living links	2-12-09
id	ID boards	Information board	A total of eight ‘identification boards’ of four different designs (east and west capuchins and squirrel monkeys) hanging from cables at a low level to encourage handing and use to identify individual monkeys in the enclosures	17-10-09
aa	Primate family tree	‘Interactive’ game or display	An interactive ‘primate family tree’ using a cursor controlled by a rollerball	19-04-10
bb	Research Training	‘Interactive’ game or display	An interactive quiz challenging visitors to identify Linking Link’s capuchin monkeys using a cursor controlled by a rollerball	19-04-10
cc,dd	Panpipes	‘Interactive’ game or display	A pair of ‘puzzle boxes’ originally used in experiments on social learning in chimpanzees (Whiten et al. 2005) and adapted for use by the public. The two puzzle boxes are identical, but each has a small video screen showing a different method of solving the puzzle	27-7-10 (screens added 2-8-10)

ee	How to speak squirrel monkey	Information board	Photos and short descriptions of visual displays and communication in squirrel monkeys	21-11-09
ff	Rainforests	Information board	Explaining the design of the outdoor enclosures of Living Links, based on the monkeys natural habitat	21-11-09
gg	How to speak capuchin	Information board	Photos and short descriptions of visual displays and communication in capuchin monkeys	21-11-09
